# A Novel Communication Rating Scale to Mitigate the Effect of Implicit Bias

**DOI:** 10.1001/jamanetworkopen.2025.32319

**Published:** 2025-09-17

**Authors:** Jennifer Tjia, Chengwu Yang, Julie Flahive, Kelly Harrison, Geraldine Puerto, Vennesa Duodu, Lisa A. Cooper, Olga Valdman, Janice Sabin

**Affiliations:** 1 Department of Population and Quantitative Health Sciences, UMass Chan Medical School, Worcester, Massachusetts; 2 Department of Obstetrics & Gynecology, UMass Chan Medical School, Worcester, Massachusetts; 3 Measurement, Outcome, and Design Section (MODS), UMass Chan Medical School, Worcester, Massachusetts; 4 Department of Psychiatry, University of Connecticut, Farmington; 5 School of Medicine, Johns Hopkins University, Baltimore, Maryland; 6 School of Nursing, Johns Hopkins University, Baltimore, Maryland; 7 Bloomberg School of Public Health, Johns Hopkins University, Baltimore, Maryland; 8 Department of Family Medicine, UMass Chan Medical School, Worcester, Massachusetts; 9 Department of Biomedical Informatics and Medical Education, University of Washington School of Medicine, Seattle

## Abstract

**Importance:**

Implicit bias recognition and management (IBRM) training is a promising strategy for improving clinician communication but is underevaluated.

**Objective:**

To describe the development and psychometric properties of the Respect, Empathize, Listen, Ask, Talk, and Engage (RELATE) rating scale, based on a theoretical framework of communication skills, to mitigate negative influences of implicit bias by using strategies for interrupting unconscious decision-making in patient-centered communication.

**Design, Setting, and Participants:**

In this cross-sectional study, between September 2019 and April 2022, standardized patients used the RELATE rating scale to evaluate communication skills of participating clinical trainees at an academic medical center who completed an IBRM training and engaged in simulated clinical encounters with standardized patients representing those with low socioeconomic status and African American, Latino or Hispanic, and African immigrant patients. Statistical analyses were ongoing from June 2021 to January 2024.

**Main Outcome and Measures:**

Gwet agreement coefficient (AC) was calculated using data from the 2019 to 2020 cohort year to assess standardized patient interrater reliability (IRR). To assess construct validity and internal consistency reliability, factor analysis techniques were used, and Cronbach α was calculated using data from 3 cohorts (2019-2020, 2020-2021, and 2021-2022).

**Results:**

Twenty-seven standardized patients generated 226 independent RELATE ratings for 123 consenting clinical trainees (14 family medicine residents [11.4%], 48 internal medicine residents [39.0%], and 61 doctor of nursing practice students [49.6%]; mean [SD] age, 30.4 [4.1] years; 82 [66.7%] female), who each had 2 RELATE scores. Of the 3-level item responses of whether a behavior was observed (“present,” “partial,” or “absent”), for all 19 items, at least 1 trainee (0.4%) was noted who did not complete the evaluated behavior, and all items had some responses of “partial” (range, 13 [5.8%] to 70 [31.0%]). Twelve of the 19 RELATE items (63.2%) showed either substantial or almost perfect IRR (Gwet AC, >0.60), and 6 items (31.6%) had moderate IRR (Gwet AC, 0.41-0.60). Factor analyses resulted in a final 4-factor solution with excellent model fit indices and strong factor loadings: respect (4 items), empathy (5 items), listening and talking (6 items), and engaging in partnership with patients (4 items). Each factor showed sound internal consistency (Cronbach α range, 0.64-0.77).

**Conclusions and Relevance:**

This cross-sectional study of the RELATE rating scale demonstrated high construct validity and good internal consistency in simulated clinician-patient encounters. The findings suggest that the RELATE rating scale is an efficient, theoretically consistent tool to evaluate implicit bias–management communication behavior among clinicians.

## Key Points

**Question:**

Can the Respect, Empathize, Listen, Ask, Talk, and Engage (RELATE) rating scale produce a valid, reliable, and consistent evaluation of clinician communication to reduce the negative influence of implicit bias?

**Findings:**

In this cross-sectional study of 123 resident physicians and doctor of nursing practice students in a simulated environment, psychometric assessment of the RELATE rating scale demonstrated good interrater reliability, construct validity, and internal consistency with bias-interrupting constructs of communicating with respect and empathy, balancing listening with talking, and engaging in partnership with patients.

**Meaning:**

The RELATE rating scale may inform the evaluation of implicit bias recognition and management training and its association with clinician communication skills.

## Teaser

This cross-sectional study evaluates the development and psychometric properties of a rating scale as a tool to mitigate implicit bias in patient-centered communication.

## Introduction

Patient centeredness in clinical encounters is associated with improved clinician-patient communication and health outcomes.^1^ Implicit bias is increasingly recognized as a threat to high-quality patient care and outcomes^2^ and is associated with less patient centeredness, greater verbal dominance, and lower patient rating of interpersonal care.^3^ Implicit bias recognition and management (IBRM) training has emerged as a promising strategy to mitigate the impact of implicit bias on patient care.^4,5,6,7,8^

Sukhera and Watling^9^ developed a framework for IBRM education that includes safety, knowledge, awareness, and mitigation strategies. A recent scoping review by Gleicher et al^10^ identified 90 articles on IBRM education for postgraduate training that categorized the curricula into 4 models: competence, skills-based, social contact, and critical. Some formative learning programs introduce skills to improve patient-centered communication^4,7^ and benefit from opportunities for formal practice, evaluation, and feedback in clinical simulation encounters using standardized patients.^11^ However, psychometrically sound standardized patient rating scales to evaluate patient-centered communication that mitigates the negative influences of bias are limited.^10,12^

The Roter Interaction Analysis System (RIAS) is used extensively in research to evaluate patient interactions in clinical encounters, but this labor-intensive coding system assessing nuances, utterances, and quality of clinical communication is an approach that requires extensive training.^13,14^ Development and validation of a patient-centered communication rating scale that can readily assess the efficacy of IBRM education in real time and during simulated medical encounters is needed. In simulation centers using standardized patient encounters, checklist evaluations are common,^15,16^ but many are not supported by validation evidence.^17,18,19^ In a recent study, Gonzalez et al^11^ developed a standardized patient evaluation checklist and examined the association of the checklist’s communication categories with clinicians’ implicit association test scores for medical cooperativeness and race but did not focus on the checklist’s psychometric properties.

The goal of this study was to describe the development and psychometric properties of a rating scale to evaluate communication skills that mitigate negative influences of bias. The scale is based on the core strategies for person- and relationship-centered communication developed by Cooper^20^ including Respect, Empathize, Listen, Ask, Talk, and Engage (RELATE). The RELATE-based intervention training was developed in the context of a National Institutes of Health–funded implicit bias mitigation intervention research study to train medical residents and doctor of nursing practice (DNP) trainees about health care inequities, structural racism, and IBRM strategies.^21^

## Methods

This cross-sectional study was reviewed and approved by the UMass Chan Medical School institutional review board. Participants provided written informed consent. This study followed the Strengthening the Reporting of Observational Studies in Epidemiology (STROBE) reporting guideline for cross-sectional studies.^22^

### Context of Rating Scale Development

The RELATE rating scale was developed in the context of a clinical trial that evaluated the impact of a clinician-facing IBRM program on clinical outcomes; details about the training intervention, trial, and data collection are described elsewhere.^21,23^ In brief, the trial enrolled cohorts of family medicine residents, internal medicine residents, and DNP students over 4 academic years at a public medical and nursing school. Participants completed case simulations with trained standardized patients who represented the local community of Black or African American, Latino or Hispanic, and African immigrant patient populations and patients with low socioeconomic status reliant on Medicaid. The parent trial was conducted from March 2018 to October 2022, for 4 successive cohorts of clinical trainees. The present study reports data from September 2019 through April 2022.

The core content of the training included implicit bias–management skills, knowledge about health care disparities, and implicit bias awareness and measurement using the Implicit Association Test.^21,23^ Standardized patients were assigned to specific cases without overlap (ie, 1 standardized patient could not portray 2 different cases); thus, each trainee who completed case simulations with standardized patients received multiple RELATE ratings from separate standardized patients. However, the 2018 to 2019 cohort included 4 separate standardized patient simulations over 2 days, while the other cohorts (2019-2020, 2020-2021, and 2021-2022) completed 2 case simulations on a single day. This change was made to minimize simulation burden and to accommodate COVID-19.

### Development of the RELATE Rating Scale

#### Domains

The RELATE rating scale was developed to capture the perspective of the standardized patient interacting with clinical trainees.^21^ Rating scale domains (ie, constructs) for implicit bias–management strategies (eg, emotional regulation, perspective taking, and person-centered communication) were refined based on strategies described by Cooper^20^ in prior work that proposed the RELATE mnemonic: respect, empathize, listen, ask about your assumptions, talk, and engage patients in problem solving (Box). Cooper’s^20^ framework is grounded in what is known about effective communication between clinicians and patients of different racial and ethnic groups and in the Ladder of Inference of Argyris^24^ and Senge et al^25^ about how to interrupt unconscious decision-making to make conscious decisions that are more effective. Further theoretical grounding for item domains was drawn from early evidence for bias-management skills proposed by Devine et al.^26^ These strategies include (1) individuation, which relies on preventing stereotypic inferences by obtaining specific information about group members,^27,28^ and (2) perspective taking, which involves taking the first-person perspective of the individual with whom you are trying to communicate. RELATE does not retain 2 controversial elements of Devine and colleagues’^26^ strategies, including stereotype replacement, which involves replacing a typical stereotype with a nonstereotypical idea, and counter-stereotypic imaging, which involves imagining a representative person who goes against a standard mental picture that is held in common by members of a group and that represents a prejudiced attitude^29^; these strategies were omitted because they can inadvertently perpetuate negative stereotypes.

Box. **Summary of Cooper’s^20^ Conceptual Framework and Core Strategies for Person- and Relationship-Centered Communication to Manage the Effects of Implicit Bias in Clinician-Patient Interactions**

**Respect**

Respect the humanity of the person in front of you regardless of whether you like them or agree with what they are saying

**Empathize**

Imagine yourself in the person’s shoes

**Listen**

Listen more and talk less

**Ask**

Ask yourself what assumptions you are making and whether they are based on facts about this particular person

**Talk**

Talk with people about their personal lives and get to know them as individuals

**Engage**

Engage people in problem solving and decision-making by asking their opinions about any joint activities you are considering

#### Item Development and Content Validation

The RELATE rating scale items were based on a prior community-developed assessment for use in the clinical simulation center; this prior instrument (called the Simulation-Based Community-Engaged Research Intervention for Informed Consent Protocol Testing and Training [SCRIIPTT] checklist) was validated to measure research assistants’ cross-cultural communication skills, with an informed consent process as measured in the simulation laboratory.^30^ For the RELATE rating scale, we adapted SCRIIPTT checklist items for a simulated clinical encounter addressing hypertension with patients across cultural differences. Each item was written by research staff (J.T., O.V.) to address each RELATE theoretical construct.^20^ To ensure the items were relevant, culturally respectful, and representative of the RELATE construct domains of bias-management behavior, items were reviewed by diverse content experts, including community stakeholders with lived experience of chronic disease, standardized patients, and clinical faculty.^21,23^ Following 2 rounds of feedback and adjustments, we reached consensus on 19 items. Thus, the content validity of the RELATE rating scale is supported by consensus.

#### Item Measurement Response Format

For the initial cohort (2018-2019), item response choices captured whether the behavior was observed or not. We refined item responses based on standardized patient feedback that requested (1) an additional response category to allow partial credit for each item and (2) clearer instructions on the item response assignment that included anchoring examples. This resulted in 3 possible responses (“present,” “partial,” or “absent”) for each item. Anchors were drawn from the RIAS^13,14^ and validated examples in the literature, such as the Four Habits Coding Scheme.^31,32^

#### Standardized Patient Rater Training and Interrater Reliability Assessment

Standardized patients were trained in the evaluation of clinical trainee interactions using the RELATE rating scale. The training involved a presentation of the bias-management communication skills training that the clinical trainees received and reviewing and discussing the RELATE rating scale and examples with simulation center and clinical faculty. The standardized patient training additionally asked standardized patients to watch 6 video-recorded standardized patient–clinical trainee encounters.

To assess interrater reliability (IRR), we asked observers to watch video recordings of in-person encounters between standardized patients and clinical trainees that occurred from 2019 to 2020. Observers completed the RELATE rating scale. Observers watched recordings on site at the clinical simulation center due to technical and security reasons, but this was discontinued in March 2020 due to the COVID-19 pandemic.

### Data Collection

Data for this psychometric analysis were drawn from 3 trial cohorts (2019-2020, 2020-2021, and 2021-2022) for independent RELATE records from clinician trainees who provided written informed consent for study participation. The IRR analysis used data from the 2019 to 2020 cohort; factor analyses used data from the 2019 to 2022 cohorts.

### Statistical Analysis

We used descriptive statistics to characterize RELATE item responses. Each unique standardized patient–clinical trainee video recording was watched by observers who evaluated trainee performance using the RELATE rating scale. We compared original (in-person) standardized patient ratings with observer ratings by forming all possible rating pairs for each scale item. For example, when there were 2 observers of 1 video, this provided 3 RELATE ratings (1 original and 2 observer), resulting in 3 rating pairs (eFigure 1 in Supplement 1). The observed agreement was calculated as the proportion of rating pair agreement for each item. We measured IRR for rating scale items using Gwet agreement coefficient (AC).^33^ We calculated Gwet AC instead of Cohen κ,^34^ which is unreliable when the distribution of agreement is skewed (ie, there is high agreement across items), a phenomenon known as the κ paradox.^29^ Gwet AC is a statistical measure of interrater agreement for items when there are 2 or more raters; for 2 raters, the observed agreement of Gwet AC is equal to the Cohen κ. Gwet AC values are interpreted as follows: less than 0 indicates no agreement; 0 to 0.20, slight agreement; 0.21 to 0.40, fair agreement; 0.41 to 0.60, moderate agreement; 0.61 to 0.80, substantial agreement; and 0.81 to 1.00, almost perfect agreement.

We performed a series of factor analyses to investigate the construct validity and theoretical RELATE internal structure of the scale, starting from a confirmatory factor analysis (CFA) of the original, theory-based factor structure, followed by exploratory factor analysis (EFA) to explore a new, reasonable factor structure if the initial CFA failed and, finally, another CFA to confirm the newly explored factor structure. Factors with eigenvalues of 1 or greater were extracted in the EFA,^35,36^ with a scree plot of the eigenvalues as additional support.^31^ The final CFA was implemented based on the new factor structure from the EFA. The following model-fit indices and the cutoff values for adequate fit^35,36^ were used: comparative fit index of 0.90 or greater, Tucker-Lewis index of 0.90 or greater, weighted root mean square residual (WRMR) of 1.00 or less, root mean square error of approximation (RMSEA) of 0.10 or less, and normalized χ^2^ (χ^2^ divided by *df*) of 5.0 or less.^35,37^ To assess the internal reliability of each domain or a unidimensional measure, we used Cronbach α.^38^ A Cronbach α value of 0.7, 0.8, or 0.9 is considered an indicator of adequate, very good, or excellent internal consistency reliability, respectively.^37^

Analyses were performed using Stata/SE, version 17 (StataCorp LLC) and Mplus, version 8.8 (Muthén & Muthén). Statistical analyses were ongoing from June 2021 to January 2024.

## Results

### Descriptive Statistics

Twenty-seven standardized patients generated 226 independent RELATE ratings for 123 consenting clinical trainees (14 family medicine residents [11.4%], 48 internal medicine residents [39.0%], and 61 DNP students [49.6%]; mean [SD] age, 30.4 [4.1] years; 82 [66.7%] female, 41 [33.3%] male), who each had 2 RELATE scores (eTable 1 in Supplement 1). Forty-two trainees (34.1%) were from the 2019 to 2020 cohort; 42 (34.1%), from the 2020 to 2021 cohort; and 39 (31.7%), from the 2021 to 2022 cohort. Of 25 unique standardized patient–clinical trainee video recordings, 15 (60.0%) were watched by 1 observer; 6 (24.0%), by 2 observers; and 4 (16.0%), by 4 observers who evaluated trainee performance using the RELATE rating scale. Item responses for the RELATE rating scale are shown in Table 1. Of the 3-level item responses (“present,” “partial,” or “absent”), for all items, at least 1 of the 226 observer responses (0.4%) noted that a trainee did not complete the evaluated behavior (“absent”), and all items had some “partial” responses, ranging from 13 (5.8%) to 70 (31.0%).

**Table 1.** Frequency Table of Rating Responses for the RELATE Items for 123 Trainees Who Completed Standardized Patient Simulations From 2019 to 2022

Item	Variable name	Item content	Rating responses, No. (%) (N = 226)		
			Absent	Partial	Present
1	Respect_001	Introduced themselves appropriately	1 (0.4)	33 (14.6)	192 (85.0)
2	Respect_002	Addressed the patient in a comfortable and professional manner	6 (2.7)	33 (14.6)	187 (82.7)
3	Respect_003	Maintained an open posture	2 (0.9)	27 (12.0)	197 (87.2)
4	Empathize_001	The clinician put themselves in the patient’s shoes	9 (4.0)	45 (19.9)	172 (76.1)
5	Listen_001	Allowed the patient to speak without interrupting	3 (1.3)	31 (13.7)	192 (85.0)
6	Listen_002	The patient felt heard	2 (0.9)	46 (20.4)	178 (78.8)
7	Listen_003	Clinician was really listening so the patient felt comfortable	9 (4.0)	43 (19.0)	174 (77.0)
8	Ask_001	Explored patient’s perceptions about their health and did not just make assumptions	5 (2.2)	43 (19.0)	178 (78.8)
9	Ask_002	If patient was nonadherent, explored reasons and barriers (remembered to explore social and cultural reasons)	10 (4.4)	70 (31.0)	146 (64.6)
10	Talk_001	Paused to allow patient to absorb information or ask questions	2 (0.9)	35 (15.5)	189 (83.6)
11	Talk_002	Avoided medical jargon	2 (0.9)	13 (5.8)	211 (93.4)
12	Talk_003	Began with exploring patient’s reason for visit using open-ended questions	4 (1.8)	30 (13.3)	192 (85.0)
13	Talk_004	Asked open-ended questions about medication, including adherence	1 (0.4)	36 (15.9)	189 (83.6)
14	Talk_005	Described and offered treatment options	3 (1.3)	30 (13.3)	193 (85.4)
15	Talk_006	Provided information in small chunks	3 (1.3)	26 (11.5)	197 (87.2)
16	Engage_001	Addressed patient’s questions and concerns, without minimizing	3 (1.3)	28 (12.4)	195 (86.3)
17	Engage_002	Worked with patient to explore possible diet and physical activity regimen change	13 (5.8)	46 (20.4)	167 (73.9)
18	Engage_003	Developed a plan of care together with the patient	4 (1.8)	39 (17.3)	183 (81.0)
19	Engage_004	Assessed patient’s understanding of the final care plan that was developed with patient	9 (4.0)	60 (26.6)	157 (69.5)

Abbreviation: RELATE, Respect, Empathize, Listen, Ask, Talk, and Engage.

### Interrater Reliability

Reliability assessment revealed agreement of more than 50% across raters for each item except item 9: “If nonadherent, explored reasons/barriers (remembered to explore social and cultural reasons, financial reasons, side effects, etc).” Gwet AC coefficient was greater than 0.60 (substantial or almost perfect) for 12 of the 19 items (63.2%) and was between 0.41 and 0.60 (moderate) for 6 of the items (31.6%). The worst-performing item was item 9 (agreement, 26 [35.6%]; Gwet AC [SE], 0.17 [0.08]; 95% CI, 0.02-0.33) (Table 2).

**Table 2.** Interrater Reliability for the 2019 to 2020 Cohort

Item	Variable name	Agreement, No. (%)	Gwet AC (SE) [95% CI]^a^
1	Respect_001	42 (57.5)	0.51 (0.07) [0.36-0.66]
2	Respect_002	47 (64.4)	0.59 (0.07) [0.45-0.73]
3	Respect_003	50 (68.5)	0.65 (0.07) [0.51-0.78]
4	Empathize_001	45 (61.4)	0.54 (0.07) [0.39-0.69]
5	Listen_001	65 (89.0)	0.88 (0.04) [0.79-0.97]
6	Listen_002	51 (69.9)	0.66 (0.07) [0.53-0.79]
7	Listen_003	52 (71.2)	0.67 (0.07) [0.54-0.80]
8	Ask_001	46 (63.0)	0.57 (0.07) [0.43-0.72]
9	Ask_002	26 (35.6)	0.17 (0.08) [0.02-0.33]
10	Talk_001	55 (75.3)	0.71 (0.06) [0.58-0.84]
11	Talk_002	63 (86.3)	0.84 (0.05) [0.73-0.95]
12	Talk_003	60 (82.2)	0.80 (0.05) [0.69-0.91]
13	Talk_004	54 (74.0)	0.71 (0.06) [0.59-0.83]
14	Talk_005	49 (67.1)	0.63 (0.07) [0.50-0.77]
15	Talk_006	44 (60.3)	0.54 (0.07) [0.40-0.69]
16	Engage_001	64 (87.7)	0.86 (0.05) [0.76-0.96]
17	Engage_002	67 (78.1)	0.75 (0.06) [0.64-0.87]
18	Engage_003	51 (69.9)	0.63 (0.07) [0.49-0.79]
19	Engage_004	47 (64.4)	0.55 (0.09) [0.39-0.71]

Abbreviation: AC, agreement coefficient.

^a^ Benchmark scale for Gwet AC is less than 0 for no agreement; 0 to 0.20, slight agreement; 0.21 to 0.40, fair agreement; 0.41 to 0.60, moderate agreement; 0.61 to 0.80, substantial agreement; and 0.81 to 1.00, almost perfect agreement.

### Construct Validity and Internal Reliability

The original, theory-based, 6-factor structure fit our data adequately, as shown by the CFA results (Table 3). However, this 6-factor structure has a single-item domain, empathy, that does not allow the assessment of its internal reliability. In addition, the eigenvalue scree plot elbow occurred at the fourth factor (eFigure 2 and eTable 2 in Supplement 1). The EFA results suggested 2 new factor structures with 4 and 5 factors (Table 3). The results of CFA on these 2 new factors indicated that the 4-factor structure fit better than the 5-factor structure, with excellent model-fit indices (normalized χ^2^, 1.243; RMSEA, 0.033 [95% CI, 0.013-0.047]; comparative fit index, 0.983; Tucker-Lewis index, 0.980; SRMR, 0.078) (Table 4). Also, since it is more desirable to have fewer factors in applied research, we decided on the 4-factor structure for our data.

**Table 3.** Confirmatory Factor Analysis Results Using the 226 Responses on the RELATE Rating Scale

Item	Variable name	6 Factors		5 Factors		4 Factors	
		Dimension name	Factor loading	Dimension name	Factor loading	Dimension name	Factor loading
1	Respect_001	Respect	0.64	Respect	0.63	Respect	0.63
2	Respect_002	Respect	0.86	Respect	0.85	Respect	0.85
3	Respect_003	Respect	0.88	Respect	0.87	Respect	0.87
4	Empathize_001	Empathy	1.00	Empathy	0.75	Empathy	0.75
5	Listen_001	Listen	0.70	Ask	0.74	Listen and talk	0.70
6	Listen_002	Listen	0.83	Empathy	0.84	Empathy	0.84
7	Listen_003	Listen	0.81	Empathy	0.82	Empathy	0.82
8	Ask_001	Ask	0.73	Empathy	0.73	Empathy	0.73
9	Ask_002	Ask	0.69	Empathy	0.69	Empathy	0.69
10	Talk_001	Talk	0.70	Engage	0.72	Engage	0.72
11	Talk_002	Talk	0.35	Listen	0.33	Listen and talk	0.34
12	Talk_003	Talk	0.72	Ask	0.76	Listen and talk	0.72
13	Talk_004	Talk	0.74	Ask	0.77	Listen and talk	0.73
14	Talk_005	Talk	0.80	Engage	0.81	Engage	0.81
15	Talk_006	Talk	0.61	Listen	0.59	Listen and talk	0.61
16	Engage_001	Engage	0.90	Engage	0.92	Engage	0.92
17	Engage_002	Engage	0.56	Respect	0.62	Respect	0.62
18	Engage_003	Engage	0.86	Engage	0.88	Engage	0.88
19	Engage_004	Engage	0.73	Listen	0.70	Listen and talk	0.72

Abbreviation: RELATE, Respect, Empathize, Listen, Ask, Talk, and Engage.

**Table 4.** Model Fit Indices for the Confirmatory Factor Analysis Results Using the 226 Responses on the RELATE Rating Scale

Factors, No.	χ^2^	*df*	Normalized χ^2^^a^	RMSEA (90% CI)	Comparative fit index	Tucker Lewis index	Weighted RMR
6	179.047	138	1.297	0.036 (0.018-0.051)	0.98	0.976	0.078
5	179.304	142	1.263	0.034 (0.015-0.049)	0.982	0.978	0.077
4	181.442	146	1.243	0.033 (0.013-0.047)	0.983	0.980	0.078

Abbreviations: RELATE, Respect, Empathize, Listen, Ask, Talk, and Engage; RMSEA, root mean square error of approximation; RMR, root mean square residual.

^a^ χ^2^ divided by *df*.

In this final model, respect and empathize items loaded as expected under each domain. Listen and talk items loaded with multiple different domains, including empathy, listen, ask, talk, and engage (Table 3). Ask items loaded onto the empathy domain. The 4-factor solution removed ask items as a separate domain. Ask, in the RELATE theoretical model, was about the clinician checking their biases and not making assumptions. In the RELATE rating scale, it was operationalized as asking the patient to share their perspective of their illness or reason for nonadherence. These items loaded with empathy, suggesting that these items were used to understand things from the patient’s point of view, which is the mechanism of bias checking in which clinicians put themselves in the patient’s shoes. Based on the model fit characteristic and Cooper’s original RELATE framework,^20^ we reorganized the original items into respect (4 items), empathy (5 items), listen and talk (6 items), and engage in partnership (4 items) (eFigure 3 in Supplement 1).

All items in the 4-factor model had factor loadings greater than 0.40, indicating at least a moderate correlation with their assigned factor, except for item 11 (“avoided medical jargon”) (Table 3). The internal consistency α coefficient was calculated for each of the 4 factors to determine their utility as subscales. Factor 1 had internal reliability (Cronbach α) of 0.68, factor 2 had internal reliability of 0.77, factor 3 had internal reliability of 0.64, and factor 4 had internal reliability of 0.76.

## Discussion

In this study, the RELATE rating scale demonstrated sound psychometric properties in assessing simulated encounters between clinical trainees and standardized patients. To our knowledge, this is the first scale with good internal consistency, internal structure, and IRR to rate IBRM and person-centered communication skills in simulated clinician-patient encounters with trained standardized patients. The most closely related validated standardized patient checklist for patient-centered communication is the Georgetown Patient-Centeredness Rating Scale, which was not about racial bias but rather was developed to evaluate clinician interactions with female patients who experienced trauma.^19^ Thus, the RELATE rating scale fills an important gap in medical, nursing, and clinical education.

Examination of the item responses across the sample indicated that the scale had adequate item distribution. The findings from our analyses provide support for the internal structure and construct validity of the RELATE scale. Specifically, the model-fit statistics supported a 4-factor rating scale that corresponded to the theoretical constructs of the original RELATE model but reorganized to operationally combine empathy and ask questions in a way that allows the evaluation of the steps by which one can develop and show empathy.^39^

Bolstering the soundness of the RELATE scale is that each of the 4 RELATE domains also corresponds with the RIAS, a patient-physician communication assessment tool that is widely used across various medical disciplines, patient and clinician populations, and medical conditions but is not often used in clinical simulation settings.^13,14^ The RIAS requires trained coders to analyze communication at the level of utterance^13^ and focuses on verbal and nonverbal communication, using descriptive narrative prompts to assist the evaluator. While the RELATE rating scale also focuses on verbal and nonverbal communication, it is user friendly and does not require in-depth evaluator training to deploy.

The RELATE rating scale provides an analysis of clinical communication that yields actionable areas of strengths and deficits in medical communications, enabling individuals and programs to tailor further skills-building training and bias-management strategies. For example, if a medical interaction assessment with the rating scale demonstrates a lack of rapport building, either generally or with a specific patient population, training could be precisely tailored to the area of deficit.

Use of the RELATE rating scale to evaluate educational interventions has the potential to improve clinical training in nearly real time by identifying specific communication behaviors that are strong as well as those that need improvement. Of note, the RELATE-based training intervention prompts trainees to increase awareness of bias, to be more deliberate about engaging in self-regulation that may reduce bias in clinical decision-making, and to promptly use communication skills that improve patient outcomes. It is most relevant for its intended role in formative learning-stage feedback in clinical education based on standardized patient case simulations. It is a tool that could enhance IBRM by quantifying communication skills in the areas of respect, empathic listening, talking skills, and engagement and partnering.^9,20^

While the RELATE rating scale was developed for standardized patient evaluations, in the future, it could be used more broadly by different types of raters in IBRM trainings (eg, for self- or peer assessment in role plays for IBRM trainings). Closely related is the use of the rating scale for summative evaluation and not just formative assessments in testing skill competencies; such an application is possible with further psychometric evaluation and rigorous standardized patient training in the standardized application of the item responses, as is common in high-stakes testing.

### Strengths and Limitations

A strength of this research is our model of collaborative input and the wisdom of diverse clinical partners, which is valuable to this emerging field. However, our findings need to be considered in the context of several limitations. First, this study was conducted at a single site and may not generalize to all sites. The standardized patients were focused on a few particular patient subpopulations, which further limits generalizability. While the clinical participants were limited to residents engaged in graduate medical education and DNP students, we believe the RELATE rating scale could also be applied to undergraduate medical education (ie, medical students) and postgraduate continuing medical education (ie, attending physicians) as well as to other health professionals. Future work needs to examine the rating scale’s concurrent and predictive validity with other indicators of patient-reported communication quality and patient outcomes. Despite these limitations, to our knowledge this is the first validation of a clinical communication evaluation tool in a field early in its development.

## Conclusions

In this cross-sectional study of the RELATE rating scale to evaluate bias-management skills in patient-centered communication, we found that the RELATE rating scale demonstrated sound psychometric properties, including high construct validity and good internal consistency in simulated encounters. The findings suggest that the RELATE rating scale is an efficient, theoretically consistent tool to evaluate implicit bias–management communication behavior among clinicians. The RELATE rating scale is an important development for the field of IBRM education because its use in program evaluation can inform precision IBRM education development as the field evolves.

## Notes

Supplement 1. **eTable 1.** Characteristics of Participants, by Cohort Year **eTable 2.** Initial Factors and Their Eigenvalues From Exploratory Factor Analysis **eFigure 1.** Schematic of Rating Pairs Resulting From 2 Observers of 1 Original Standardized Patient-Trainee Encounter **eFigure 2.** Scree Plot of Eigenvalues From the Exploratory Factor Analysis **eFigure 3.** Final RELATE Standardized Patient Rating Scale Items

Supplement 2.
Data Sharing Statement
