# Supplementary material for: A Novel Communication Rating Scale to Mitigate the Effect of Implicit Bias
Source: JAMA Netw Open. 2025 Sep 17;8(9):e2532319. doi: 10.1001/jamanetworkopen.2025.32319 (PMC12444548; doi:10.1001/jamanetworkopen.2025.32319)
Supplement: Supplement 1. — eTable 1. Characteristics of Participants, by Cohort Year eTable 2. Initial Factors and Their Eigenvalues From Exploratory Factor Analysis eFigure 1. Schematic of Rating Pairs Resulting From 2 Observers of 1 Original Standardized Patient-Trainee Encounter eFigure 2. Scree Plot of Eigenvalues From the Exploratory Factor Analysis eFigure 3. Final RELATE Standardized Patient Rating Scale Items [file jamanetwopen-e2532319-s001.pdf]

Supplementary Online Content

Tjia J, Yang C, Flahive J, et al. A novel communication rating scale to mitigate the effect of implicit bias. *JAMA Netw Open*. 2025;5(4):e214732. doi:10.1001/jamanetworkopen.2021.4732

eTable 1. Characteristics of Participants, by Cohort Year
eTable 2. Initial Factors and Their Eigenvalues From Exploratory Factor Analysis
eFigure 1. Schematic of Rating Pairs Resulting From 2 Observers of 1 Original Standardized Patient-Trainee Encounter
eFigure 2. Scree Plot of Eigenvalues From the Exploratory Factor Analysis
eFigure 3. Final RELATE Standardized Patient Rating Scale Items

This supplementary material has been provided by the authors to give readers additional information about their work.

© 2025 Tjia J et al. *JAMA Network Open*.

eTable 1. Characteristics of Participants, by Cohort Year

Cohort	Family Medicine Residents N (%)	Internal Medicine Residents N (%)	Doctor of Nursing Practice (DNP) Students N (%)	Total N=123
2019-20	5 (11.9)	16 (38.1)	21 (50.0)	42
2020-21	5 (11.9)	19 (45.2)	18 (42.9)	42
2021-22	4 (10.3)	13 (33.3)	22 (56.4)	39

© 2025 Tjia J et al. *JAMA Network Open*.

eTable 2. Initial Factors and their Eigenvalues from Exploratory Factor Analysis

Factor	Eigenvalue	Difference	Proportion	Cumulative
Factor 1	6.32	4.94	0.33	0.33
Factor 2	1.38	0.04	0.07	0.41
Factor 3	1.34	0.28	0.07	0.48
Factor 4	1.06	0.003	0.06	0.53
Factor 5	1.06	0.12	0.06	0.59

© 2025 Tjia J et al. *JAMA Network Open*.

eFigure 1. Schematic of Rating Pairs Resulting from Two Observers of One Original Standardized Patient-Trainee Encounter

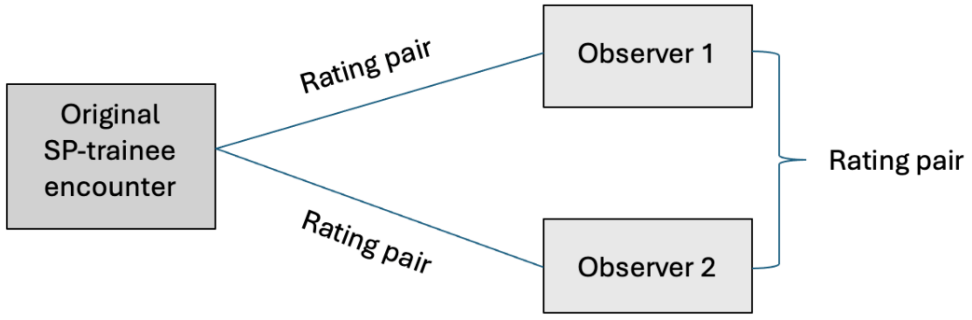

© 2025 Tjia J et al. *JAMA Network Open*.

eFigure 2. Scree Plot of Eigenvalues from the Exploratory Factor Analysis

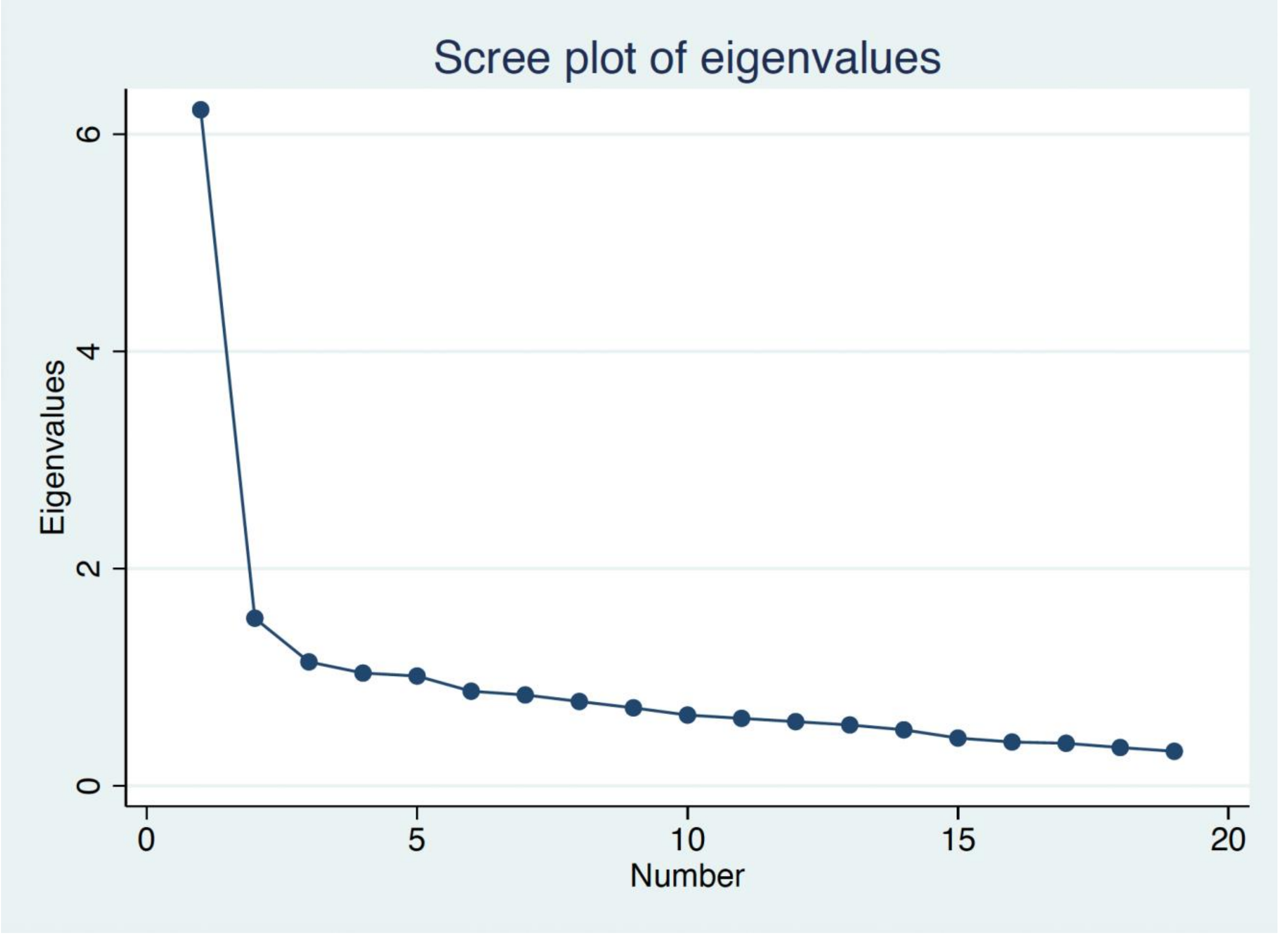

© 2025 Tjia J et al. *JAMA Network Open*.

eFigure 3. Final RELATE Standardized Patient Rating Scale Items

	**Community-Engaged Simulation Training RELATE Rating Scale Items**	**No 1**	**Partial 3**	**Yes 5**
	**Respect (4 items)**			
1	**Introduced themselves appropriately**	Greet warmly[1]		
	• Patient is greeted in manner that is personal and warm (e.g., clinician asks patient how they like to be addressed, uses patient's name).			X
	• Patient is greeted in manner that recognizes patient, but without great warmth or personalization.		X	
	• Greeting of patient is cursory, impersonal, or nonexistent.	X		
2	**Addressed the patient in a comfortable and professional manner**	Small talk[1]		
	• Clinician makes non-medical comments, using these to put the patient at ease (e.g. asks about what is happening in my daily life or with my family).[2]			X
	• Clinician makes cursory attempt at small talk (shows no great interest, keeps discussion brief before moving on).		X	
	• The clinician gets right down to business without any attempt at small talk (or cuts patient off curtly and abruptly, or if later in visit, shows only passing interest).	X		
3	**Maintained an open posture**	Show good nonverbal behavior[1]		
	• Clinician displays nonverbal behaviors that express great interest, concern and connection (e.g., eye contact, tone of voice, and body orientation) throughout the visit.			X
	• Clinician's nonverbal behavior shows neither great interest or disinterest (or behaviors over course of visit are inconsistent).		X	
	• Clinician's nonverbal behavior displays lack of interest and/or concern and/or connection (e.g., little or no eye contact, body orientation or use of space inappropriate, bored voice).	X		
4	**Worked with patient to explore possible diet and physical activity regimen changes**	Involve in decisions[1]		
	• Clinician clearly encourages and invites patient's input into the decision-making process.(e.g. Let's work together to come up with a plan for lowering your blood pressure)			X
	• Clinician shows little interest in inviting the patient's involvement in the decision-making process, or responds to the patient's attempts to be involved with relatively little enthusiasm.		X	
	• Clinician shows no interest in having patient's involvement or actively discourages/ignores patient's efforts to be part of decision-making process.	X		
	**Empathy (5 items)**			
5	**The health care clinician puts themself in the patient's shoes**	Can view things from my perspective (see things as I see them)[1]		
	• Clinician makes statements that paraphrase, name or recognize the emotional state of the other person during the visit. (e.g. I can see that there are a lot of things going on for you right now that make it really difficult for you to go out and exercise.)			X
	• Clinician briefly acknowledges patient's feelings but makes no effort to indicate acceptance/validation. (e.g. A universal, "I understand", but not in a caring way)		X	

© 2025 Tjia J et al. *JAMA Network Open*.

	• Clinician shows no interest in patient’s emotional state and/or discourages or cuts off the expression of emotion by the patient (signals verbally or nonverbally that it is not okay to express emotions).	X		
**6**	**The patient felt concerns were “heard”**	Expansion of concerns[1]		
	• The clinician encourages the patient to expand in discussing his/her concerns (e.g., using various continuers such as “Aha, tell me more”, “Go on.”)			X
	• Clinician neither cuts the patient off nor expresses great interest in learning more (listens, but does not encourage expansion or further discussion).		X	
	• The clinician interrupts or cuts the patient off in his/her attempt to expand (is clearly not very interested).	X		
**7**	**Health care provider was really listening so patient felt comfortable sharing underlying story/truth**	Impact on life[1]		
	• Clinician attempts to determine in detail/shows great interest in how the problem is affecting patient’s lifestyle (work, family, daily activities).[2]			X
	• Clinician attempts to determine briefly/shows only some interest in how the problem is affecting patient’s lifestyle.		X	
	• Clinician makes no attempt to determine/shows no interest in how the problem is affecting patient’s lifestyle.	X		
**8**	**Explored patient’s perception about their health, didn’t just make assumptions**	Patient’s understanding of problem[1]		
	• Clinician shows great interest in exploring the patient’s understanding of the problem (e.g., asks the patient what the symptoms mean to them).[2]			X
	• Clinician shows brief or superficial interest in understanding the patient’s understanding of the problem.		X	
	• Clinician makes no attempt/shows no interest in understanding the patient’s perspective.	X		
**9**	**If non-adherent, explored reasons/barriers (remembered to explore social and cultural reasons, financial reasons, side-effects, etc.)**	Explore barriers[1]		
	• Clinician fully explores barriers to implementation of treatment plan.			X
	• Clinician briefly explores barriers to implementation of treatment plan.		X	
	• Clinician does not address whether barriers exist for implementation of treatment plan.	X		
	**Listen and Talk (6 items)**			
**10**	**Allowed patient to speak without interrupting**	Full Agenda[1]		
	• The clinician attempts to elicit the full range of the patient’s concerns by generating an agenda early in the visit (clinician does other than simply pursue first stated complaint).			X
	• The clinician makes some reference to other possible complaints, or asks briefly about them before pursuing the patient’s first complaint, or generates an agenda as the visit progresses.		X	
	• The clinician immediately pursues the patient’s first concern without an attempt to discover other possible concerns of the patient.	X		
**11**	**Avoided medical jargon**	Gives clear explanations[1]		
	• Information is stated clearly and with little or no use of jargon			X
	• Information contains some jargon and is somewhat difficult to understand.		X	
	• Information is stated in ways that are technical or above patient’s head (indicating that the patient has probably not understood it fully or properly).	X		
**12**	**Began with exploring patient’s reason for visit using open-ended questions**	Goals for Visit[1]		

© 2025 Tjia J et al. *JAMA Network Open*.

	• Clinician asks (or responds with interest) about what the patient hopes to get out of the visit (e.g., can be general expectations or specific requests such as meds, referrals).			X
	• Clinician shows interest in getting a brief sense of what the patient hopes to get out of the visit, but moves on quickly.		X	
	• Clinician makes no attempt to determine (shows no interest in) what the patient hopes to get out of the visit.	X		
13	**Asked open-ended questions about medication, including adherence**	Question style[1]		
	• The clinician tries to identify the problem(s) using primarily open-ended questions (asks questions in a way that allows patient to tell own story with minimum of interruptions or closed ended questions).			X
	• The clinician tries to identify the problem(s) using a combination of open and closed ended questions (possibly begins with open-ended but quickly reverts to closed ended).		X	
	• The clinician tries to identify the problem(s) using primarily closed-ended questions (staccato style).	X		
14	**Provided information in small chunks**	Allow time to absorb[1]		
	• Clinician pauses after giving information with intent of allowing patient to react to and absorb it.			X
	• Clinician pauses briefly for patient reaction, but then quickly moves on (leaving the impression that the patient may not have fully absorbed the information).		X	
	• Clinician gives information and continues on quickly with giving patient opportunity to react (impression is that this information will not be remembered properly or fully appreciated by the patient).	X		
15	**Assessed patient's understanding of the final care plan that was developed with patient and health care clinician**	Test for comprehension[1]		
	• Clinician effectively tests for the patient's comprehension.			X
	• Clinician briefly or ineffectively tests for the patient's comprehension.		X	
	• Clinician makes no effort to determine whether the patient has understood what has been said.	X		
**Engage in partnership (4 items)**				
16	**Paused to allow patient to absorb information or ask questions**	Encourages questions[1]		
	• Clinician openly encourages and asks for additional questions from patient (and responds to them in at least some detail).			X
	• Clinician allows for additional questions from patient, but does not encourage question asking nor respond to them in much detail.		X	
	• Clinician makes no attempt to solicit additional questions from patient or largely ignores them if made unsolicited.	X		
17	**Described and offered treatment options**	Offers rationale[1]		
	• Clinician fully/clearly explains the rationale behind current, past, or future tests and treatments so that patient can understand the significance of these to diagnosis and treatment.			X
	• Clinician only briefly explains the rationale for tests and treatments.		X	
	• Clinician offers/orders tests and treatments, giving little or any rationale for these.	X		
18	**Addressed and legitimized patient's questions AND concerns, without minimizing**	Uses patient frame of reference[1]		
	• Clinician frames diagnostic and other relevant information in ways that reflect patient's initial presentation of concerns.[2]			X
	• Clinician makes cursory attempt to frame diagnosis and information in terms of patient's concerns.		X	

© 2025 Tjia J et al. *JAMA Network Open*.

	• Clinician frames diagnosis and information in terms that fit physician's frame of reference rather than incorporating those of the patient.	X		
**19**	**Developed a plan of care together with the patient**	Explore plan acceptability[1]		
	• Clinician explores acceptability of treatment plan, expressing willingness to negotiate if necessary.			X
	• Clinician makes brief attempt to determine acceptability of treatment plan, and moves on quickly.		X	
	• Clinician offers recommendations for treatment with little or no attempts to elicit patient's acceptance of (willingness or likelihood of following) the plan.	X		

FOOTNOTES

[1] Responses anchored to Krupat et al, The Four Habits Coding Scheme: Validation of an instrument to assess clinicians' communication behavior, Pat Ed Counsel 2006; 62: 38-45.

[2] Example from the Jefferson Scale of Patient Perceptions of Physician Empathy from Hojat M, DeSantis J, Gonnella JS. Patient Perceptions of Clinician's Empathy: Measurement and Psychometrics. J Patient Exp. 2017 Jun;4(2):78-83. doi: 10.1177/2374373517699273. Epub 2017 Mar 20. PMID: 28725866; PMCID: PMC5513637.

© 2025 Tjia J et al. *JAMA Network Open*.
