# Supplementary material for: A Novel Communication Rating Scale to Mitigate the Effect of Implicit Bias
Source: JAMA Netw Open. 2025 Sep 17;8(9):e2532319. doi: 10.1001/jamanetworkopen.2025.32319 (PMC12444548; doi:10.1001/jamanetworkopen.2025.32319)
Supplement: Supplement 2. — Data Sharing Statement [file jamanetwopen-e2532319-s002.pdf]

Data Sharing Statement

Tjia. A Novel Communication Rating Scale to Mitigate the Effect of Implicit Bias. *JAMA Netw Open*. Published September 17, 2025. doi:10.1001/jamanetworkopen.2025.32319

Data
Data available: No

Additional Information
Explanation for why data not available: Data used in this study were provided under informed consent of the participants and with strict understanding that the research data will not be shared outside of the research team.
